# The Evolution of the Major Hepatitis C Genotypes Correlates with Clinical Response to Interferon Therapy

**DOI:** 10.1371/journal.pone.0006579

**Published:** 2009-08-11

**Authors:** Phillip S. Pang, Paul J. Planet, Jeffrey S. Glenn

**Affiliations:** 1 Department of Medicine, Division of Infectious Diseases and Geographic Medicine and Division of Gastroenterology and Hepatology, Stanford University School of Medicine, Palo Alto, California, United States of America; 2 Department of Pediatrics, Division of Infectious Diseases, Columbia Presbyterian Medical Center and, Sackler Institute of Comparative Genomics, American Museum of Natural History, New York, New York, United States of America; 3 Department of Medicine, Division of Gastroenterology and Hepatology, Stanford University School of Medicine and the Palo Alto Veterans Administration Medical Center, Palo Alto, California, United States of America; National Institute for Communicable Diseases, South Africa

## Abstract

**Background:**

Patients chronically infected with hepatitis C virus (HCV) require significantly different durations of therapy and achieve substantially different sustained virologic response rates to interferon-based therapies, depending on the HCV genotype with which they are infected. There currently exists no systematic framework that explains these genotype-specific response rates. Since humans are the only known natural hosts for HCV–a virus that is at least hundreds of years old–one possibility is that over the time frame of this relationship, HCV accumulated adaptive mutations that confer increasing resistance to the human immune system. Given that interferon therapy functions by triggering an immune response, we hypothesized that clinical response rates are a reflection of viral evolutionary adaptations to the immune system.

**Methods and Findings:**

We have performed the first phylogenetic analysis to include all available full-length HCV genomic sequences (n = 345). This resulted in a new cladogram of HCV. This tree establishes for the first time the relative evolutionary ages of the major HCV genotypes. The outcome data from prospective clinical trials that studied interferon and ribavirin therapy was then mapped onto this new tree. This mapping revealed a correlation between genotype-specific responses to therapy and respective genotype age. This correlation allows us to predict that genotypes 5 and 6, for which there currently are no published prospective trials, will likely have intermediate response rates, similar to genotype 3. Ancestral protein sequence reconstruction was also performed, which identified the HCV proteins E2 and NS5A as potential determinants of genotype-specific clinical outcome. Biochemical studies have independently identified these same two proteins as having genotype-specific abilities to inhibit the innate immune factor double-stranded RNA-dependent protein kinase (PKR).

**Conclusion:**

An evolutionary analysis of all available HCV genomes supports the hypothesis that immune selection was a significant driving force in the divergence of the major HCV genotypes and that viral factors that acquired the ability to inhibit the immune response may play a role in determining genotype-specific response rates to interferon therapy.

## Introduction

Nearly 170 million people worldwide are chronically infected with hepatitis C virus (HCV) [1]. In the US, HCV is the leading cause of hepatocellular carcinoma and the leading indication for liver transplantation [2]. The standard of care for the treatment of chronic hepatitis C is combination therapy with pegylated interferon and ribavirin. Pegylated interferon (PEG-IFN) is a synthetic variant of interferon-α, a naturally occurring cytokine whose endogenous role is to activate the innate immune response. Injected PEG-IFN is hypothesized to function by mimicking this natural cytokine. Ribavirin (RBV) is a nucleoside analog. It is thought to act through a combination of modalities (as reviewed in [3], [4]).

Large clinical trials of PEG-IFN/RBV therapy have revealed significantly different response rates for the various HCV genotypes. There are six major HCV genotypes, numbered 1 to 6. Genotype 2 is the most responsive, with a sustained virologic response (SVR) rate of greater than 80%. Studies also suggest that it is reasonable to treat some patients infected with this genotype for only 12–16 weeks [5], [6], [7]. Conversely, the most prevalent genotype worldwide, genotype 1, is the least responsive. The SVR rate for patients infected with genotype 1 is less than 50%. Current guidelines recommend 48 weeks of therapy for this genotype; shorter courses of therapy have been demonstrated to be sub-optimal [8].

There currently exists no systematic explanation for these genotype-specific differences in clinical outcome [4], [9], [10]. It is assumed that genotype-specific clinical response rates are the result of a confluence of host and viral factors. What specific host factors, human demographics/geographic patterns, and/or viral factors determine interferon response rates remains a challenging area of inquiry. Furthermore, whether factors that govern outcome for one genotype play a similar role in other genotypes remains to be more fully explored.

Numerous laboratory studies suggest that certain viral factors are able to inhibit aspects of the innate immune response (as reviewed in [11], [12], [13]). These cell culture studies, however, highlight the gap that currently exists between laboratory models and the human host. For example, the HCV replicon system allows for the study of HCV RNA replication in cell culture. Using this system, it was observed that genotype 2 replicons were more resistant to interferon than genotype 1 replicons, the opposite of what is observed clinically [14]. Thus, this observation may be a culture system artifact [15] that highlights the challenge of ascertaining the clinical relevance of findings first discovered in laboratory models.

Humans are the only known natural hosts for HCV, a virus that is estimated to be hundreds and possibly thousands of years old [16], [17], [18]. This lengthy relationship may have allowed HCV to accumulate adaptive mutations that confer increasing resistance to the human immune system. Interferon therapy functions by activating the innate immune response, which is comprised of direct intracellular defenses such as the PKR, Mx and RNaseL proteins, and innate immune cells, including NK, dendritic, monocyte, macrophage, and NKT cells. Once activated, the innate immune system also plays a critical role in the proper stimulation and coordination of the adaptive immune response [13], [19].

We therefore hypothesize that genotype-specific clinical response rates to interferon-based therapies are a reflection of HCV evolutionary adaptations to the immune system. We do not hypothesize that modern interferon therapy itself selected for the various HCV genotypes. Instead, we are hypothesizing that the immune system that is activated by interferon therapy has co-evolved with HCV.

One evolutionary pattern that would strongly indicate that a selective pressure was favoring adaptations to the immune system would be a strict correlation of increasing non-response to treatment with the relative ages of the genotypes—such that, as each new genotype emerged it would have a more resistant phenotype than its ancestor.

**Figure 1:**
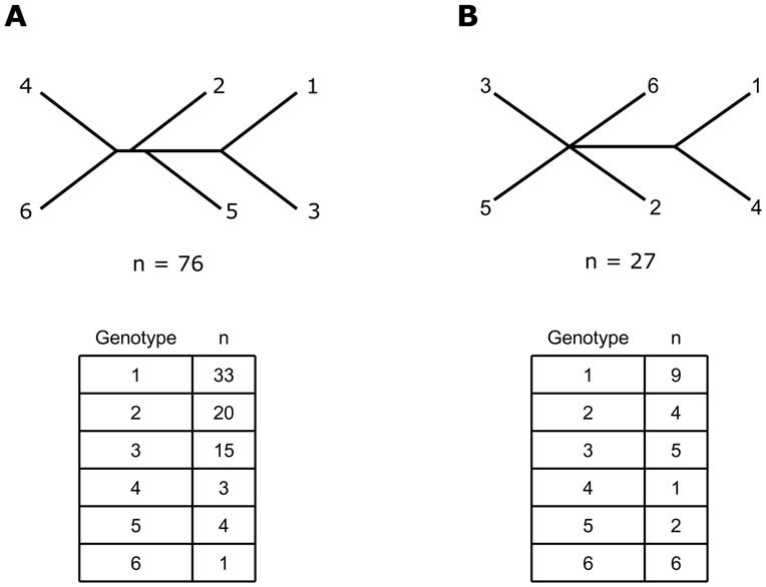
Unrooted HCV Cladograms From Previous Studies. Panel A shows the first cladogram to divide HCV into six genotypes, based on a neighbor-joining analysis of the NS5 region of HCV that included 76 sequences (Simmonds *et al.* 1993). Panel B shows a more recent HCV consensus tree with a different genotype branching pattern compared to Panel A, based on an analysis of 27 full-length HCV genomic sequences (Salemi *et al.* 2002). The table below each panel indicates the genotype distribution of the sequences analyzed in these studies.

HCV was first divided into genotypes by the seminal work of Simmonds and others in 1993 [20], [21], based on an analysis of one segment of the HCV genome from 76 different patients (Figure 1A). Evolutionary analysis limited to only portions of a genome, however, can be misleading [18], [21], [22]. For instance, by analyzing 27 full-length HCV genomes Salemi and colleagues [23] (Figure 1B) found a different phylogenetic pattern for the relationships amongst the six HCV genotypes. Also of note is that neither analysis determined the relative evolutionary ages of the various genotypes.

Determining the relative ages of the major HCV genotypes is critical to testing our hypothesis that a correlation exists between genotype age and clinical resistance. Relative ages can be determined through the use of an outgroup, which roots the phylogeny and establishes the direction of time. In the analysis presented here, we used GB Virus B (GBV-B) as an outgroup. This allowed us to root our HCV phylogeny and establish for the first time the relative ages of the major HCV genotypes. GBV-B was chosen as the outgroup for two reasons: first, GBV-B, a virus that causes hepatitis in New World monkeys, is the closest viral relative of HCV and the only other member of the *hepacivirus* genus. Second, biochemical evidence suggests that proteins in GBV-B share highly-specific functionality with their homologs in HCV [24].

The evolutionary analyses of HCV that have been performed to date have also been based on a limited number of genomic sequences. A prime reason for this is that reliable methods of tree construction, such as maximum likelihood (ML) and maximum parsimony (MP), require considerable amounts of computational power. Thus, often only a subset of available sequences is actually analyzed. For this reason, Salemi *et al*. limited their analysis to 27 genomes.

In this work, we perform a comprehensive analysis of all the >300 genomes found in the European HCV database. This order of magnitude increase in the number of genomes analyzed was made possible by the NSF-funded Cyberinfrastructure for Phylogenetic Research (CIPRES) Project, which allows for web-based access to the San Diego Super Computer facility and newly developed evolutionary algorithms that dramatically reduce computational time.

We thus sought to construct the first evolutionary tree of HCV that incorporated all known genomic sequences and would allow for the relative ages of all HCV genotypes to be determined. We then used this tree to test our hypothesis that clinical resistance to interferon correlates with HCV genotype age. Finally, we used ancestral sequence reconstruction to identify HCV loci that potentially play a role in determining genotype-specific clinical outcomes.

## Methods

### Sequence Selection and Alignment

All 348 full-length HCV genomic sequences publicly available as of October 2007, were downloaded from the European HCV database [25]. Reflecting its wider prevalence, 236 of these sequences were genotype 1. Forty-five sequences were genotype 2, seven were genotype 3, ten were genotype 4, two were genotype 5, and forty-five were genotype 6. Three sequences were putative recombinants and were discarded.

Two separate methods were used to align the coding regions of these sequences, in order to address potential alignment strategy biases. The first method utilized ClustalX [26] followed by inspection, which ensured that the alignment respected known viral protein properties. The second alignment method, MAFFT [27], was fully automated. Over 90% of the HCV genome encodes a single polyprotein. To ensure in-frame alignment, amino acid sequence alignments were first generated and then used to guide the nucleic acid sequence alignments. For both alignment programs, gap opening and extension parameters were set to their defaults.

GBV-B was selected as the outgroup because it is the closest known viral relative of HCV that is not thought to fall within the HCV clade [24], [28], [29]. Broadening the outgroup to include GB virus A and GB virus C was not possible because the alignment of these viruses to HCV was poor. Thus, these viral sequences were not used in our analyses.

### Phylogenetic Analyses

**Figure 2:**
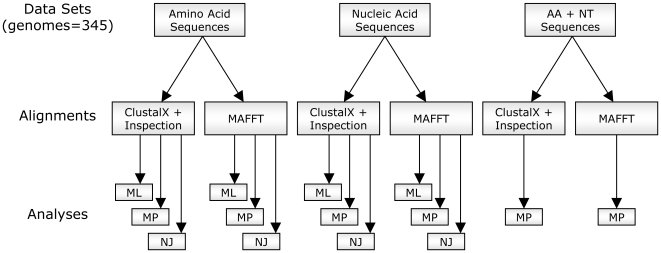
Flow Chart of the Evolutionary Analyses Performed in This Study. ML: maximum likelihood; MP: maximum parsimony; NJ: neighbor joining; MAFFT: multiple sequence alignment based on fast Fourier transform.

Our two amino acid alignments and the two nucleotide (NT) alignments, each alignment having been generated by the two alignment methods described above, were then analyzed using ML, MP, and neighbor-joining (NJ) techniques (Figure 2). Additionally, a combined alignment was created by concatenating our amino acid and nucleotide datasets into a single matrix. This combined alignment was then analyzed using the MP method. Combining amino acid sequences with nucleotide sequences has been proposed as a method for extracting as much information from sequences as possible and acting to weigh protein coding amino acid data without *a priori* transformation costs [30].

PAUP [31] was used to perform the NJ analyses. The minimum evolution criterion was used and branch lengths were allowed to be negative except when calculating tree scores. In this situation, branch lengths were set to zero. Ties were broken randomly. The best NJ tree (amino acid dataset) score was −9.09196; the best NJ tree (nucleotide dataset) score was −12.83397.

For MP analyses, the program PAUPRat [32] in conjunction with PAUP [31] was used to perform an aggressive search using the Ratchet method [33]. 200 ratchet iterations were performed for each dataset with random addition (RA) followed by tree branch reconnection (TBR) swapping, randomly upweighting 15% of characters at each iteration and saving only one tree at each iteration. The resulting trees were then used as starting trees for TBR searching using the Multrees option in PAUP. Gaps were treated as a state and all characters and state transformations were weighted equally. Bootstrap values were calculated using 100 bootstrap iterations, using 10 replicates of RA followed by TBR in each iteration [29]. The data set for each iteration was generated by re-sampling (with replacement) the characters in the alignment. Bremer supports [29] were calculated using the program Autodecay [34] in conjunction with PAUP, using 10 TBR replicates for each node in the MP phylogeny. See Table 1 for MP tree scores and statistics.

**Table 1:** Parsimony Tree Statistics.

	Optimal Score (Clustal)	No. of Trees Found (Clustal)	Consistency Index	Retention Index	Rescaled Consistency Index	Consensus Fork Index	Rohlf Consensus Index	Total No. of Characters (Parsimony Informative)
**AA+NT**	175615	228	0.162	0.76	0.123	0.901	0.806	12881 (8107)
**NT**	140497	32	0.125	0.752	0.094	0.977	0.959	9669 (6227)
**AA**	3334	31104	0.293	0.799	0.234	0.936	0.94	3212 (1880)

ML NT analyses were performed using GARLI [35], via the CIPRES portal [36]. Prior ML analyses used a GTR+Gamma site model [37]. We used the program HYPHY [38] to perform both exhaustive and hierarchical model testing based on the likelihood ratio, and determined the optimal model to be GTR+gamma+invariant. Nevertheless, use of invariant sites did not affect the tree structure determined (data not shown). The shape of the gamma function was inferred from the data set. More than 35 separate ML analyses were performed, in order to address stochastic concerns and better ensure that the resultant tree had the optimal maximum likelihood. The optimal NT ML tree -lnL score was 594309. RAxML[39] was used to assess node support with 100 rapid bootstrap replicates. Bootstrap values were drawn on the best-scoring GARLI ML. RAxML was also used for amino acid analyses, using a WAG+Gamma+F model. The optimal AA ML tree -lnL score was 198250. At the time of our analyses, the CIPRES server used RA with no Multrees and no swapping for heuristic tree searching. Out of concern that this less aggressive search strategy might limit our ML analyses, we also used as starting trees the outputs from our more aggressive MP search strategy. Resultant trees were unchanged.

### Evaluating Taxon Sampling

All available whole HCV genomes were initially analyzed, based on the assumption that adding taxa increases phylogenetic accuracy [40]. As a consequence, genotype 1 sequences represented 236/345 of the analyzed genomes. We utilized two approaches to address the potential bias introduced by this uneven taxonomic sampling. First, we performed our analyses using three different optimality criteria, since uneven taxon sampling might be expected to cause the various optimality criteria to produce different phylogenetic trees [41], [42]. Second, we utilized a taxon jackknifing technique. In our jackknife analyses, genotype 5 sequences were excluded, as only two existed. Each jackknifed dataset was constructed by randomly selecting seven taxa per genotype, without replacement, from the initial alignments. The number seven was selected as this represented the number of taxa in genotype 3, the second least represented genotype. Random selection was performed 10 times, for each of the amino acid, nucleotide and concatenated alignments, resulting in 30 datasets, each of which consisted of 35 taxa plus the outgroup GBV-B. All of the above described analyses were then repeated on these datasets.

### Clinical Trial Data Compilation

A PubMed search for English language, prospective trials that studied the effect of PEG-IFN/RBV combination therapy for the treatment of chronic hepatitis C was used to identify relevant trials. Published reviews were also consulted to ensure comprehensiveness [8], [43]. Studies published (including early e-publication) up to December 2007 were included. The fact that sustained virologic response rates vary according to genotype has already been well-established [5], [8], [43], [44], [45], [46]. For illustrative purposes, were therefore compiled here only the major, large prospective trials. Specifically, early trials with less than 100 patients in total or arms with less than 25 patients were excluded, except in the case of genotype 4 for which there primarily exist a limited number of smaller trials. For genotype 4, trials with less than 25 patients were excluded. Published non-inferiority trials comparing different PEG-IFN formulations were also excluded, as they assessed only end-of-treatment virologic response. The genotype-specific clinical response rates shown are based on intention-to-treat analyses, and represent averages weighted according to the number of patients in each indicated study (Table 2). Notably, clinically relevant differences in outcome among HCV sub-genotypes (subtypes) [8] have not been observed. Our approach was therefore limited to genotype-level outcome data.

**Table 2:** Prospective HCV Trials of Therapy with Pegylated Interferon and Ribavirin.

Genotype	Avg. SVR	Therapy Duration	Patients	Study Specific SVR^a^	
**1**	**46%**	**48 wks**	917	Hadziyannis *et al*. 2004 [44]	52% (141/271)
				Fried *et al*. 2002 [102]	46% (137/298)
				Manns *et al*. 2001 [45]	42% (145/348)
**2**	**82%**	**12–24 wks**	788	Dalgard *et al.* 2008^b^ [103]	97% (30/31)
				Shiffman *et al.* 2007^b^ [6]	75% (268/356)
				Yu *et al.* 2007 [7]	95% (142/150)
				Mangia *et al.* 2005 [5]	80% (171/213)
				Von Wagner *et al.* 2005 [104]	92% (35/38)
**3**	**72%**	**12–24 wks**	657	Dalgard *et al*. 2008^b^ [103]	92% (106/115)
				Shiffman *et al.* 2007^b^ [6]	66% (244/369)
				Mangia *et al.* 2005 [5]	66% (46/70)
				Von Wagner *et al.* 2005 [104]	73% (75/103)
**4**	**60%**	**24–48 wks**	676	Kamal *et al*. 2007 [46]	63% (239/378)
				Derbala *et al*. 2005 [105]	29% (10/35)
				Kamal *et al*. 2005 [106]	70% (48/69)
				El-Zayadi *et al.* 2005 [107]	55% (22/40)
				Alfaleh *et al*. 2004 [108]	43%(12/28)
				Hasan *et al.* 2004 [109]	68% (45/66)
				Shobokshi *et al*. 2003^c^	50% (30/60)
5	*No prospective trials of pegylated interferon and ribavirin*				
6	*No prospective trials of pegylated interferon and ribavirin*				

a Sustained Virologic Response: Number of patients with no detectable virus 6 months after completion of therapy divided by the total number of patients treated, based on intention-to-treat analyses.

b Data from the 24 week arm of the study.

c 2003 AASLD Abstract # 996.

### Testing Correlation

Standard tests of correlation are not appropriate for items that are related by descent in an evolutionary hierarchy [47], [48]. We therefore used Shimodaira's approximately unbiased (AU) tree-based statistical test [49], [50] to evaluate the critical nodes within our tree topology and test the statistical significance of our observed correlation between relative genotype age and SVR. Specifically, we constrained the clade including genotypes 1 and 4 not to exist in our optimal tree (T_no1,4_). We then constrained the clade including genotypes 1, 3, 4, and 5 to not exist in our optimal tree (T_no1,4,3,5_). Each of these alternate trees, which disrupt the evolutionary branching pattern of the major genotypes, was then tested against the optimal ML tree using Shimodaira's AU test with the RELL bootstrap approximation (1000 replicates), to determine if they were statistically inferior. These analyses were carried out using the program CONSEL [51].

### Identification of Viral Resistance Loci

Fitch optimization [52] was used to reconstruct the ancestral protein sequence at each of the three nodes that lie on the main trunk of our tree [53], [54]. These ancestral sequences were then screened, in two steps, to identify the positions that correlate with increased resistance. First, because resistance adaptations result from sequence mutation, these ancestral sequences were screened to identify residues that had undergone mutation as HCV evolved along the main trunk of the tree. These positions were then screened to identify positions that, having mutated, conserved the particular mutation in all future progeny. This second screening step is based on the assumption that functionally advantageous mutations are likely to be conserved.

## Results

**Figure 3:**
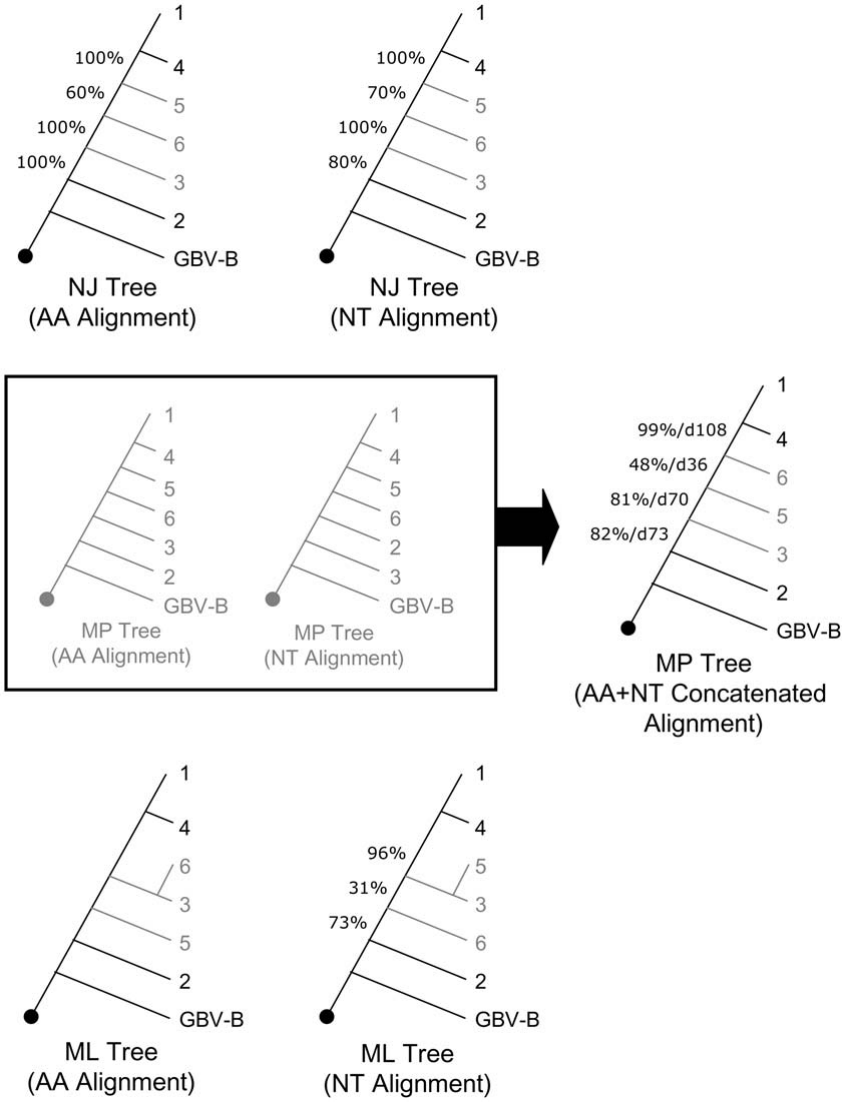
Rooted Neighbor-Joining (NJ), Maximum Parsimony (MP), and Maximum Likelihood (ML) cladograms depicting the evolution of the major hepatitis C virus genotypes. A nexus file of complete tree data is available online (Dataset S1). Numbers represent bootstrap support and Bremer decay indices. AA: amino acid sequences; NT: nucleotide sequences.

The results of each individual phylogenetic analysis are shown in Figure 3. Regardless of whether amino acid or nucleotide sequence data was analyzed, or which of the two alignment methods were utilized, striking concordance was found to exist among the three phylogenetic inference methods. Individual optimal trees from each analysis were also supported by bootstrap values and/or Bremer decay indices, and differed only in the placement of genotypes 5 and 6. In all but one case (parsimony nucleotide analysis) the relative branching order of those genotypes with prospective clinical outcome data (i.e. genotypes 1, 2, 3, and 4) was identical.

**Figure 4:**
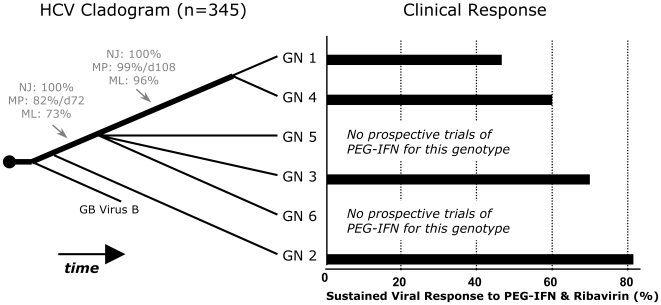
Rooted Consensus Cladogram Resulting From An Analysis of 345 Full-Length HCV Genomic Sequences. The evolution of the major (HCV) genotypes and their correlation to clinical outcome is depicted. Values denote bootstrap support and Bremer decay indices for the indicated phylogenetic inference method.

The end result of our phylogenetic analyses is summarized in figure 4, left panel. It reveals that HCV genotype 2 branched first, genotypes 1 and 4 branched last, and genotypes 3, 5, and 6 branched sometime after genotype 2 but before genotype 4. Using population genetic methods to analyze limited portions of the genome from a sampling of genotypes (1, 4, and 6), Pybus and colleagues [16], [18] estimated origin times for genotypes 1, 4 and 6 that are notably concordant with the more complete branching order we have determined. Other studies [17], [18], [55] have inferred the absolute age of certain HCV sub-genotypes/subtypes (e.g. 1a, 1b and 3a) and found them to be relatively recent; these finding do not contradict the relative ages we have inferred for the major genotypes.

To gauge the effect of taxon sampling on our phylogenetic analysis, we performed the taxon jackknife technique described above, in which we randomly selected an equal number of taxa from each genotype for repeated analyses. All jackknife replicates using the amino acid data, for all optimality criteria (ML, NJ, and MP), gave exactly the same genotype branching order seen in Figure 4. For nucleotide data, jackknife replicates analyzed using NJ and MP methods also resulted in the same branching order. Likewise, the combined amino acid and nucleotide datasets analyzed using MP criteria were 100% concordant with the overall consensus. The ML analyses of the jackknifed nucleotide data sets resulted in inconsistent branching patterns. Only one ML NT jackknifed data set reslted in a tree with the same branching pattern as seen in our overall consensus. The remaining nine differed amongst each other in the location of the root and/or overall topology, with most rooting within or near genotype 6.

A compilation of the outcome data from 19 prospective trials of combination therapy with PEG-IFN/RBV to treat chronic hepatitis C is shown in Table 2. As has been previously observed [5], [8], [43], [44], [45], [46], genotype-specific response rates are hierarchical. From highest to lowest, the pattern of SVR rates is: genotype 2>genotype 3>genotype 4>genotype 1. There currently exist no prospective trials of PEG-IFN/RBV therapy for genotypes 5 and 6. Notably, no compelling evidence exists that suggests that sub-genotypes (i.e. 1a versus 1b) have clinically relevant differences in outcome [8], though evidence exists that sequence variations within subtypes may affect clinical outcome [56], [57]. Therefore, the present study was restricted to a genotype-level analysis of clinical response and sequence evolution.

When genotype-specific clinical response rates were mapped onto our phylogenetic tree, a correlation between genotype age and clinical resistance was revealed (Figure 4). As hypothesized, early branching genotypes were noted to have the best clinical outcomes and require the least duration of therapy, while genotypes that branched later (i.e. more recently) have higher rates of clinical resistance and need to be treated for much longer. Therefore, each newly emerged genotype has greater resistance than its ancestor, indicating the likely presence of a selective pressure favoring resistance. To test the statistical significance of this correlation between relative genotype age and SVR, we applied Shimodaira's AU test (see Methods). Briefly, we searched for the best possible trees that disrupted the observed branching pattern and compared their likelihoods to the likelihood of our optimal tree. In every case, trees that contradicted our tree topology and disrupted the observed correlation with interferon susceptibility were significantly suboptimal (P<0.01). Thus, by this rigorous standard, the observed correlation is statistically significant. A far more conservative estimate of the significance of this correlation was calculated using the equation, N = (2n−3)!/2n−2(n−2)!, where n is the number of taxa (in this case, genotypes are the taxa), and N equals the number of ways in which taxa can be ordered on a branching tree. Thus there were a total of N = 15 possible ways in which the 4 genotypes with known clinical outcomes could be ordered on our tree, making the likelihood of a tree pattern that matched SVR outcomes, purely by chance, equal to 1 out of 15.

This correlation between clinical resistance and branching order allows for the following prediction: prospective clinical trials of genotypes 5 and 6 using PEG-IFN/RBV, for which prospective data is currently lacking, will likely show intermediate SVR rates, similar to genotype 3. Antaki and colleagues have published a retrospective study of genotype 5 infected patients treated with PEG-IFN/RBV. By this retrospective analysis, genotype 5 infected patients have an SVR rate of 67% [58], exactly as our tree predicts.

**Figure 5:**
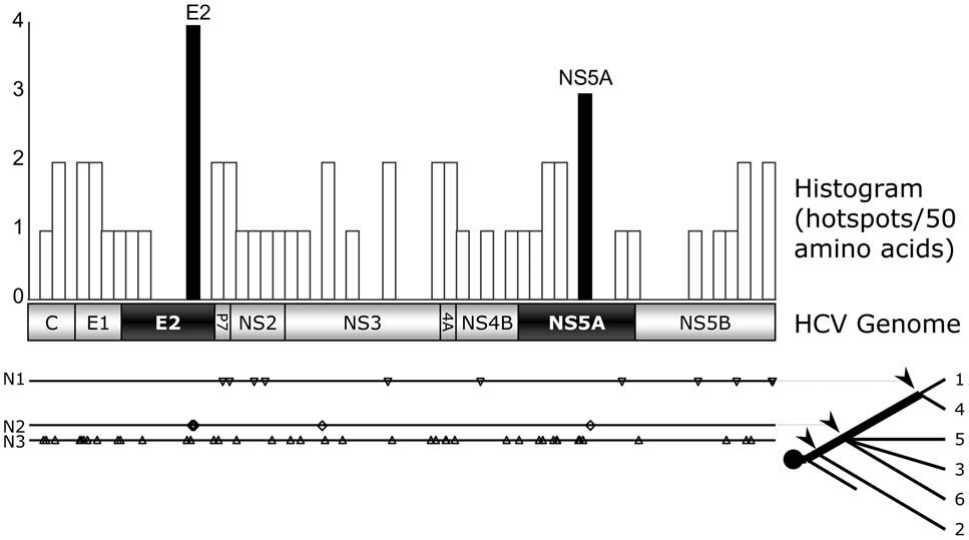
HCV Resistance Loci. Positions (“hotspots”) in the HCV genome that appear to have undergone directed change with respect to immune resistance. N1: positions associated, using ancestral sequence reconstruction techniques, with genotype (GN) 1 being the most resistant. N2: positions associated, using ancestral sequence reconstruction techniques, with GNs 1 and 4 being more resistant than GNs 2, 3, 5, and 6. N3: positions associated, using ancestral sequence reconstruction techniques, with GNs 1, 3, 4, 5, and 6 being more resistant than GN 2. A hotspot histogram (binned in groups of 50 amino acids) is shown at the top. The 10 proteins encoded by the HCV genome are also illustrated. Two loci were identified with the highest density of hotspots (black bars); these loci map to the PePHD domain of E2 and the PKR binding domain of NS5A, both of which have been shown to inhibit the innate immune factor PKR.

Ancestral sequence reconstruction, as described in Methods, was then used to identify viral elements potentially responsible for this clinical resistance trend. Our analysis resulted in 55 hot spots: positions that mutated as HCV became increasingly resistant. The locations of these hotspots are shown in Figure 5. N1 denotes positions associated, by reconstruction analysis, with genotype 1 being more resistant than any other genotype; N2 denotes positions associated with genotypes 1 and 4 being more resistant than genotypes 2, 3, 5, and 6; N3 denotes positions associated with genotypes 1, 3, 4, 5, and 6 being more resistant than genotype 2.

These hotspots were then binned in groups of 50 amino acids, resulting in the frequency histogram shown at the top of Figure 5. This histogram identifies the two 50 amino acid regions of the HCV genome that contain the greatest number of hotspots. These two regions fall within the PKR binding domains of the E2 and NS5A proteins of HCV [59], [60], suggesting their potential importance as viral factors that may determine genotype-specific responses to interferon therapy.

## Discussion

In this study, the relative evolutionary age of HCV genotypes has been shown to correlate with the likelihood of a sustained virologic response to interferon-based therapies (Figure 4). We suggest that the observed correlation stems from consistent selective pressure generated by the host immune system. However, a number of alternative explanations could account for the observed correlation between response to interferon-based therapies and relative genotype age. It is possible that the observed correlation is a product of chance, bias, or analytical limitations. Alternatively, the branching pattern may be accurate, but the patterns may not be directly causally related. We discuss each of these possibilities below, as well as the current experimental evidence in favor of immune-mediated selection.

With regard to chance, bias, or potential analytical limitations, our results are dependent on the reliability of evolutionary reconstruction techniques and limitations of the data set. We have attempted to address these concerns through the use of three different techniques (NJ, ML, and MP), two alignment methods, and rigorous statistical testing. Our approach included performing bootstrap and Bremer decay index analyses to measure support for branches. Importantly, we note that neither the small discrepancies that exist between phylogenetic techniques, nor areas of weaker support, contradict the overall branching pattern of the tree.

As many available whole HCV genomes as possible were analyzed based on the assumption that adding taxa increases phylogenetic accuracy [40]. However, since genotype 1 has been more well-sampled than other genotypes, this assumption may have introduced a bias due to uneven taxonomic sampling. Such uneven taxon sampling might be expected to cause the various optimality criteria used here to produce different phylogenetic trees [41], [42]; however, we have shown that they essentially do not. One could further argue that all phylogenetic optimality criteria may have been affected by uneven taxon sampling bias in the same way, leading each to converge on the wrong answer. To address this possibility, we utilized a taxon jackknifing technique, in which an equal number of taxa were randomly selected from each of the genotypes for analysis. This random selection process was repeated 10 times for each type of dataset. For all dataset types, for all inference methods (except the ML analyses of the NT datasets), and for all jackknife replicates, exactly the same genotype branching pattern was obtained. Thus, the observed genotype branching pattern appears robust both to reducing the number of taxa and to equalizing the number of genotype representatives.

Sparse taxon sampling, as opposed to uneven taxon sampling, may have resulted in greater uncertainty of branch placement for less represented genotypes, such as genotype 5, for which only 2 full-length sequences were available. This uncertainty (as reflected in figure 3), led to the inability to determine a definitive relationship of genotype 5 to genotypes 3 and 6. We could only determine that it branched after genotype 2, and before genotypes 1 and 4.

The above tests control for the possibility that the branching pattern of the tree itself was the result of analytical limitations, bias, or chance. To gauge the validity of the observation that this branching order correlates with clinical response to interferon, we used a battery of comparisons based on Shimodaira's AU test. Notably, the AU test showed that alternative tree topologies that disrupt the observed correlation are statistically highly unlikely.

With regard to whether this correlation is the result of a causal relationship, we note that our data satisfies a number of criteria commonly used to address the plausibility of causality in statistical analyses (i.e. the Hill criteria [61]). First, we show a biological gradient of responsiveness that correlates with increasing genotype age, with all four genotypes that have well-defined clinical outcomes adhering to this pattern (Figure 4). Second, ancestral sequence reconstruction was used to identify viral proteins putatively involved in genotype-specific immune resistance. These same proteins have been independently identified in biochemical analyses [36], [37] to have the ability to inhibit the innate immune response. Thus, the observed correlation has mechanistic plausibility and is consistent with other evidence. Third, biochemical experimentation has been able to reproduce some of the biological gradient we predict. The ability of the E2 and NS5A proteins to inhibit PKR is genotype-specific and consistent with our evolutionary tree [59], [60]. An analysis of host cleavage of viral RNA by the RNaseL defense pathway is also consistent with our observed trend [62], [63]. For these reasons, it is reasonable to hypothesize that a causal relationship explains our observations, while recognizing that further experimental evidence is needed.

### Possible Mechanisms of Immune Selection

Our phylogenetic results are consistent with our hypothesis that a selective pressure generated by the host immune system has played a significant role in HCV evolution and the divergence of genotypes. The specific combination of interferon-induced immune pathways responsible for this evolution, however, remains largely a matter of speculation.

For example, given that interferon can cause an up-regulated, multi-specific, HCV-specific CD4+ T-cell response [64], one possibility is that genotype-specific differences in clinical outcome are the result of differences in antigenicity [65]. Patients who progress to chronic infection compared to those that resolve their acute infection have increased viral diversity in their envelope gene E2. Thus, diversity may be a product of the emergence of antibody-selected escape variants [66]. HCV escape variants selected by CD8+ T cells, which recognize other regions of the virus, have also been observed [67], [68], [69], [70]. It remains to be determined, however, whether these observed micro-evolutionary processes are the same macro-processes that produced genotype divergence.

An alternative and non-mutually exclusive possibility is that HCV genotype-specific differences in clinical outcomes are the result of the ability to cause immune dysfunction. Patients with up-regulated interferon stimulated genes (ISG) prior to therapy are likely to be non-rapid responders [71], [72]. One possible interpretation of this data is that HCV causes immune dysfunction. ISGs are up-regulated but non-functioning or uncoordinated in their response [12]. Notably, an up-regulated ISG state was found to be more prevalent in more refractory genotypes, such as genotype 1 and 4, than 2 and 3 [72].

Yet another possibility is that genotype-specific outcomes are the result of direct inhibition of the intracellular immune pathways within liver cells. One key interferon intracellular pathway activates double-stranded RNA-dependent protein kinase (PKR). PKR shuts down protein production in infected cells, preventing them from being used as factories for virus production. Biochemical and cell culture studies have determined that HCV proteins E2 and NS5A are capable of inhibiting PKR activation [59], [60]. Notably, the motif of E2 that we identified as having key ancestral sequence changes that correlated with genotype immune differentiation is the same region identified by biochemical analysis to be critical for E2 inhibition of PKR–the PKR-eiF2α phosphorylation homology domain (PePHD). Critically, the ability of E2 to inhibit PKR was found to be specific to genotype 1 [59], the genotype most strongly associated with non-response to therapy [8]. Similarly, our ancestral sequence reconstruction analysis identified the same NS5A locus determined by biochemical studies to be important for NS5A inhibition of PKR (PKR binding domain) [60]. Shimotohno and colleagues have observed that the ability of NS5A to inhibit PKR is genotype specific: NS5A from genotype 1 is able to inhibit the innate immune factor PKR, but the NS5A protein from genotype 2 cannot [73]. This biochemical observation can now be explained by our evolutionary analysis.

Another defense mechanism activated by interferon is the RNaseL pathway. When activated, RNaseL cleaves viral RNA. Evidence suggests that genotype 1 is the most resistant of all genotypes to RNaseL, while genotypes 2 and 3 are these least resistant [62], [63]. Such differences in RNaseL susceptibility are likely mediated by differences in nucleotide composition. This data is also consistent with our observed correlation between relative genotype age and clinical responsiveness. Notably, a number of other viral factors in HCV that help it overcome the immune response (as reviewed in [12], [13]), have been described. The vast majority of these studies, however, involved only HCV genotype 1. Extending such studies to other genotypes would shed much light on the immunobiology of this virus. Such studies may also eventually reveal that PKR inhibition and RNaseL evasion are only a subset of the genotype-specific defense mechanisms that have evolved as a result of immune selection.

### Origins of Current HCV Diversity

Our results raise an important secondary question: Why have the more interferon sensitive genotypes (e.g. genotype 2) not become equally resistant over time, especially given the high mutation rate of HCV? At least four, non-mutually exclusive possibilities might explain a pattern in which certain descendants have a beneficial phenotype while other relatives do not.

First, the beneficial trait could have evolved in a specific historical epidemiological context. Given the phylodynamic pattern of HCV [74], this is quite possible. The opportunity for early HCV genotypes to acquire certain traits may have already passed.

Second, the beneficial trait could be relatively difficult to acquire from a genetic perspective. Although HCV has a high mutation rate, a high mutation rate does not necessarily imply that certain traits will be easily acquired (as reviewed in detail by Smith and Simmonds [75]). For HCV specifically, immune resistance may be difficult to acquire due to structure-functional restrictions on the mutagenic space that current HCV strains can explore. Studies have shown that all of the HCV non-structural proteins interact with one another [76]. Mutation is thus limited to non-critical regions or would require multiple, simultaneous compensatory mutations that preserve function. Mutation space in HCV is further constrained by genome ordered RNA structure (GORS) [77]. Significant RNA structure has been found to underlie the entire HCV polyprotein, which would further limit the ability of HCV to mutate without disrupting critical structure-function relationships. [65], [75].

The existence of structure-function limitations on the genetic diversity of HCV is clear from the fact that mutations in HCV are not homogeneously distributed [65], [69], [75], [78], [79], [80]. Mutations are over-represented in defined hypervariable regions such as the N-terminal half of E2, which is where antibodies have been found to bind [66], [81]. Conversely, the C-terminal region of E2 that we and others identify as significant for genotype-specific inhibition of PKR is quite static [82], [83], [84].

The available clinical data also provides indirect support for the possibility that resistance to interferon is not easily acquired over the time scale of treatment. To date, the most likely causes for therapeutic failure are non-compliance, insufficient drug levels or dose reductions, drug toxicity, and interruptions in therapy–not resistant, escape variants [85], [86]. In fact, a study of the sequence variability in the NS5A gene during treatment found no selection of interferon resistant HCV strains [87]. Another study of the full length polyprotein also found no significant difference in the number of mutations between non-responders and relapsers [88]. Although accelerated mutational change with interferon treatment was observed to occur in another study, such changes were notably not related to treatment duration and therefore were not felt to explain treatment non-response [89]. The available clinical evidence, therefore, does not contradict the possibility that resistance to interferon may be genetically difficult to acquire.

A third possible reason why different susceptibilities still exist is that resistance to the immune system may come at some cost or interfere with some other selective advantage. It is possible that the earlier branching genotypes possess specialized functions that we do not yet fully recognize, which compensate for their lack of ability to interfere with the immune system. This possibility is supported by reports of clinical differences in the course of disease for different genotypes [90], [91], [92].

Fourth, it is possible that early branching genotypes maintain higher susceptibilities because of host immune heterogeneity—i.e. through diversifying selection. Numerous host factors have been shown to effect clinical outcome [93]. One example is the observation that specific host NK-KIR receptor-HLA combinations have been shown to be a determinant of whether acute HCV infection resolves or progresses to chronic infection [94]. A second example of host heterogeneity is the observation that the binding of interferon to its cellular receptor triggers a far more attenuated transcriptional response in African-Americans compared to Caucasians [95]. The SVR pattern of HCV may be a product of all of these phenomena.

### Complexities of HCV Biology and Evolution

We suggest that the human immune system has played a significant role in shaping the evolution of HCV. However, it seems highly unlikely that the evolution of HCV has been driven by a single factor. In fact, other studies have emphasized demographic and spatial history as the predominant forces in HCV evolution [74]. This may explain why genotype 6 is endemic to South East Asia while genotype 4 is endemic to central Africa and the Middle East [18], [65], [96], [97], [98]. Such phylogeographic explanations are not necessarily inconsistent with our results. Epidemiological processes and immune selection may both have played a role in HCV evolution. For instance, spread of HCV between host populations with inherent immune differences could have been a major evolutionary determinant.

We also acknowledge that this evolutionary analysis does not explain why the treatment of acute HCV is genotype independent [99], [100]. We can only speculate that when acutely infected patients are treated, their disease may not yet have established a firm foothold, and that treatment overwhelms any viral inhibition abilities. It may also be the case that chronic infection allows for viral dissemination to more immune-protected regions of the host [101].

In conclusion, the observed correlation between relative genotype age and the probability of a sustained virologic response supports our hypothesis that immune selection played a role in HCV genotype level divergence. Such a correlation suggests the intriguing forecast that prospective clinical trials for genotypes 5 and 6 will show intermediate response rates, between genotypes 4 and 2, to interferon-based regimens. This prediction is notably in line with current retrospective data regarding the SVR for genotype 5 [58]. It also highlights the need for more molecular studies that explore the genotype-specificity of HCV-immune interactions. Finally, this hypothesis provides a systematic framework to explain, and by which to explore, the molecular nature of non-responsiveness to clinical treatment for HCV.

## Supporting Information

Dataset S1 Nexus file of select phylogenetic trees
(0.07 MB TXT)
